# The inner membrane complex through development of *T**oxoplasma gondii* and *P**lasmodium*

**DOI:** 10.1111/cmi.12285

**Published:** 2014-03-21

**Authors:** Clare R. Harding, Markus Meissner

**Affiliations:** ^1^ Wellcome Trust Centre for Molecular ParasitologyInstitute of Infection, Immunity and InflammationCollege of Medical, Veterinary and Life SciencesThe University of GlasgowGlasgowUK

## Abstract

*P**lasmodium* spp. and *T**oxoplasma gondii* are important human and veterinary pathogens. These parasites possess an unusual double membrane structure located directly below the plasma membrane named the inner membrane complex (IMC). First identified in early electron micrograph studies, huge advances in genetic manipulation of the Apicomplexa have allowed the visualization of a dynamic, highly structured cellular compartment with important roles in maintaining the structure and motility of these parasites. This review summarizes recent advances in the field and highlights the changes the IMC undergoes during the complex life cycles of the Apicomplexa.

## Introduction

The large and diverse infrakingdom of single‐celled eukaryotes termed Alveolates possess a highly specialized endomembrane system found directly beneath the plasma membrane (Adl *et al*., 2007; Gould *et al*., 2008). In the apicomplexan parasites, causative agents of a number of medically and economically devastating diseases, this structure is referred to as the inner membrane complex (IMC) (Morrissette and Sibley, 2002). The IMC has a number of important roles in the complex life cycles of these parasites, including providing structural stability, as an important scaffold in daughter cell development and as the location of the actin‐myosin motor complex, a key component in parasite motility and host cell invasion. Recently, understanding of the structure and components of the IMC has significantly increased with the recognition of various subdomains within the IMC (Beck *et al*., 2010; Poulin *et al*., 2013) and its dynamic composition throughout cell division and maturation (Anderson‐White *et al*., 2011; Kono *et al*., 2012).

This review will focus on the role and composition of the IMC through development of two of the best‐studied Apicomplexa, *Plasmodium* spp., the causative agent of malaria and *Toxoplasma gondii*, the cause of Toxoplasmosis.

## The structure of the inner membrane complex (IMC)

The inner membrane complex is made up of flattened membrane sacs termed alveoli, supported on the cytoplasmic face by a highly organized network of intermediate filament‐like proteins termed the subpellicular network (SPN) (Mann and Beckers, 2001; Kudryashev *et al*., 2010) and by interactions with the microtubule cytoskeleton (Dubremetz *et al*., 1979; Morrissette *et al*., 1997).

In *T. gondii* tachyzoites and bradyzoites, the IMC is composed of three rows of fused rectangular vesicles encircling the parasite with openings at the apical and basal ends (Porchet and Torpier, 1977; Dzierszinski *et al*., 2004; Del Carmen *et al*., 2009). A similar arrangement is seen in *Plasmodium* gametocytes where the IMC is formed from between 9 and 15 plates (Meszoely *et al*., 1982; Kono *et al*., 2012). However, in all other *Plasmodium* life stages, the IMC is formed from a single fused vesicle (Dubremetz *et al*., 1979; Bannister and Mitchell, 1995; Raibaud *et al*., 2001; Kono *et al*., 2012).

## Alveoli

Although the flattened membrane vesicles are a crucial component of the IMC, comparatively little is known beyond their function as an anchor for IMC‐resident proteins. The luminal contents of the alveoli have not been defined in apicomplexans. However, in the alveolate *Paramecium*, alveoli have been demonstrated to act as calcium stores (Ladenburger *et al*., 2009) and it has been suggested that this may also be the case in *Plasmodium* (Holder *et al*., 2012) and presumably *T. gondii*. The mobilization of calcium from this source remains to be demonstrated, although the localization of calcium‐dependent kinases between the IMC and plasma membrane in both *Plasmodium* and *T. gondii* may support this theory (Billker *et al*., 2009).

Although a number of proteins localized to the IMC are now known, comparatively little is known about the lipid content of the alveoli membranes. Previously it has been demonstrated that large areas of the IMC membranes in *T. gondii* are resistant to detergent extraction due to a high concentration of cholesterol (Coppens and Joiner, 2003; Johnson *et al*., 2007). These areas appear responsible for the immobilization of the actin‐myosin motor complex (Johnson *et al*., 2007) and are potentially linked to the presence of TgNCR1 in the IMC, a cholesterol‐binding protein involved in lipid metabolism (Lige *et al*., 2011). Interestingly, the IMC‐localized protein TgHsp20 was shown to bind to the phosphoinositides (PtdIns) PtdIns(4)P and PtdIns(4,5)P_2,_ demonstrating that these lipid species are present in the alveolar membrane (de Miguel *et al*., 2008; Coceres *et al*., 2012). The lack of uniform TgHsp20 staining on the IMC (de Miguel *et al*., 2008), suggests either that the alveoli contain subdomains defined by varying PtdIns compositions, or that other IMC‐associated proteins block TgHsp20 recruitment to the whole IMC surface. This irregular Hsp20 staining can also be visualized in *Plasmodium* sporozoites where PbHsp20 appears important for motility and is re‐localized to the tips of sporozoites during gliding (Montagna *et al*., 2012a).

The membranes of the alveoli are home to a number of proteins, some of which have now been defined. Recently, a novel family of proteins termed IMC subcompartment proteins (ISPs) have been used to delineate various sub compartments of the IMC through parasite division (Beck *et al*., 2010; Poulin *et al*., 2013). In the tachyzoite stage, *T. gondii* divides by endodyogeny; the construction of two daughter cells within the mother, followed by budding of the daughter cells (reviewed in Anderson‐White *et al*., 2013; Francia and Striepen, 2014). TgISP1 localizes to the apical cap of the parasite and is one of the first markers seen at initiation of daughter cell construction. TgISP2 and TgISP4 localize to the central section of the IMC, while TgISP3 is found only at the basal end. Interestingly, disruption of TgISP2 (although not TgISP4) resulted in a severe fitness loss with parasites appearing to attempt construction of many daughter cells within a single mother (termed endopolygeny), suggesting that TgISP2 has an important role in regulating cell division in *T. gondii* (Beck *et al*., 2010; Fung *et al*., 2012). The ISP proteins are myristoylated in the cytoplasm and then, with the exception of TgISP4, palmitoylated at the developing IMC (Beck *et al*., 2010; Fung *et al*., 2012). It is thought that the palmitoylation at the membrane is responsible for the observed hierarchical localization of ISPs; however this remains to be demonstrated (Beck *et al*., 2010). Interestingly, in *Plasmodium berghei*, there is no homologue for TgISP2 or 4. Instead, disruption of PbISP1 is lethal in the asexual stages while deletion of PbISP3 results in an upregulation of PbISP1 and no discernable phenotype throughout the life cycle. These data highlight the divergent strategies used by Apicomplexa to regulate the cell cycle and demonstrates that the composition of the IMC has important roles in parasite development (Poulin *et al*., 2013).

## Subpellicular network

Beneath the alveoli lies a network of interwoven 8–10 nm filaments named the subpellicular network (SPN) which gives the parasite strength and stability (Mann and Beckers, 2001). The filaments making up this network are named alveolins, a family of intermediate filament‐like proteins conserved between all members of the infrakingdom Alveolata (Gould *et al*., 2008). Alveolins are of variable size and are characterized by multiple repeats, usually including the subrepeat motifs EKIVEVP, EVVR or VPV, flanked by highly variable amino‐ and carboxyl‐terminal regions (Gould *et al*., 2008). The first alveolin characterized in Apicomplexa was shown to localize to the SPN and named TgIMC1 (Mann and Beckers, 2001). TgIMC1 was shown to be highly resistant to detergent extraction and was post‐translationally processed very late in daughter cell budding, which appeared to result in an increased stability of the daughter cell SPN (Mann and Beckers, 2001; Mann *et al*., 2002). Identification of TgIMC1 in *T. gondii* was followed by the localization of TgIMC3 via a tagging approach (Gubbels *et al*., 2004) and TgIMC4 through proteomic analysis of the conoid (Hu *et al*., 2006). A systemic search has since discovered a total of 14 alveolin‐repeat‐containing proteins in *T. gondii*, TgIMC1 and TgIMC3–15 (Anderson‐White *et al*., 2011), TgIMC2 does not contain characteristic alveolin repeats and is not considered an alveolin (Mann and Beckers, 2001). In *Plasmodium*, eight IMC1 homologues have been named (IMC1a–h); however, others have been identified and the total number of alveolins present in the species has not yet been definitively documented (Khater *et al*., 2004; Kono *et al*., 2012).

In addition to its role in maintaining the structural stability of the parasite, an intriguing secondary role for alveolins has been suggested. Long linker molecules, apparently derived from the SPN, have been observed linking the SPN with organelles including the apicoplast, mitochondria and ER in *Plasmodium* sporozoites (Kudryashev *et al*., 2010). It is possible that the parasite uses these linkers to maintain the relative position of organelles during gliding motility. Such linkers have not yet been observed in *T. gondii* or in other *Plasmodium* species or life cycle stages, but are too fine to be visualized using conventional light microscopy. Further electron microscopy studies will be required to confirm the existence of these filaments.

## Intramembranous particles

In order for the SPN to be able to stabilize the alveoli, there must be a physical link between the two structures. This link is thought to be mediated by 9 nm intramembranous particles (IMPs) found with distinct periodicity on all four faces of the alveolar membranes in both *T. gondii* and *Plasmodium* ookinetes (Dubremetz *et al*., 1979; Morrissette *et al*., 1997; Raibaud *et al*., 2001). On the cytoplasmic face of the IMC, IMPs are seen in a double line overlaying microtubules (Morrissette *et al*., 1997) and a single line, probably following the path of the filaments making up the SPN (Mann and Beckers, 2001) which stretch across the borders of the flattened vesicular sacs. No constituents of IMPs have yet been conclusively identified; however, one potential candidate is PbG2 (identified as TgILP1 in *T. gondii*), a small, non‐alveolin protein localized to the SPN (Lorestani *et al*., 2012; Tremp *et al*., 2013). When disrupted, PbG2 was shown to be required for maintaining the morphology of ookinetes and sporozoites, in a similar manner to the alveolins PbIMC1b and PbIMC1h (Tremp *et al*., 2008; 2013; Tremp and Dessens, 2011). Interestingly, and unlike the alveolins tested, this altered morphology did not result in a decrease in the tensile strength of the IMC. This suggests that PbG2 has a discrete function from alveolins and may be mediating the interaction between the SPN and alveoli (Tremp *et al*., 2013). Another possible constituent of IMPs are the oligomeric multipass membrane proteins named GAPM1 (also identified as PfM6Tβ, PFD1110w), GAPM2 (PfM6Tγ, MAL13PI.130) and GAPM3 (PfM6Tα, PF14_0065) (Bullen *et al*., 2009; Rayavara *et al*., 2009). Interestingly, PbGAPM proteins appear to co‐immunoprecipitate with both alveolins and components of the actin‐myosin motor (Bullen *et al*., 2009), suggesting that these proteins form a direct link through the double membrane of the alveoli between the motor complex and the SPN.

## The IMC through the *P**lasmodium* life cycle

During its complex life cycle, *Plasmodium* undergoes a number of metamorphoses. These changes are associated with significant alterations in structure and function of the IMC.

In sporozoites, the IMC is essential for the localization of the actin‐myosin motor and maintenance of the structure and infectivity of the parasite (Bergman *et al*., 2003; Khater *et al*., 2004; Montagna *et al*., 2012b). The alveolin PbIMC1a is essential for maintaining the structure, tensile strength, motility and infectivity of this life cycle stage, but is redundant in other stages (Khater *et al*., 2004). After invading a hepatocyte, slender *Plasmodium* sporozoites transform into spherical trophozoites by initially bulging in the centre, followed by the retraction of the apical and basal ends (Meis *et al*., 1985; Kaiser *et al*., 2003). This metamorphosis is associated with disruption of the IMC, starting at the site of the bulge, followed by the IMC peeling away from the plasma membrane and being packaged as dense, membrane whorls in the cytoplasm (Bergman *et al*., 2003; Jayabalasingham *et al*., 2010; Poulin *et al*., 2013). These whorls of excess membrane then appear to be exocytosed through the now bare plasma membrane, along with the now unnecessary invasion organelles such as micronemes and rhoptries (Jayabalasingham *et al*., 2010).

The mature spherical trophozoite then undergoes schizogony where multiple, asynchronous rounds of mitosis are followed by the budding of merozoites from the host (for a review see Gerald *et al*., 2011). In these parasites, nuclear division is associated with IMC development in order to ensure correct organelle packaging and provide each parasite with an IMC as it emerges. In early schizonts, two structures, sometimes identified as the apical pore, containing integral IMC proteins and the acylated protein PfGAP45 can be seen close to the nucleus (Bullen *et al*., 2009; Hu *et al*., 2010; Yeoman *et al*., 2011; Ridzuan *et al*., 2012). This structure colocalizes with the centrosome marker PfCentrin3, then forms a ring which quickly extends down the nascent daughter cell, in parallel with the parasite's encapsulation by plasma membrane (Bullen *et al*., 2009; Hu *et al*., 2010; Yeoman *et al*., 2011). PfMORN1 has been shown to localize to the free ends of the developing IMC and potentially has a role in maintaining the IMC during development and later delineating the basal complex (Ferguson *et al*., 2008). Interestingly, a second set of alveolin proteins including PF3D7_0525800 (previously annotated as PFE1285w) and PF10_0039 do not localize to this early structure, and instead form a distinct ring after the initial structure is formed. These two regions remain separate until very late in schizogony when they colocalize around the budding merozoites (Kono *et al*., 2012). Once released, merozoites go on to infect new erythrocytes and the IMC is required to localize the motor complex required for invasion (Baum *et al*., 2006; Jones *et al*., 2006; Yeoman *et al*., 2011) and also likely has a role in maintaining structural stability of parasites in the bloodstream. Currently, no alveolin proteins have been identified as required for this life cycle stage; however, this may be due to the difficulties in manipulating proteins required for asexual reproduction.

During asexual reproduction, a small proportion of merozoites differentiate into gametocytes and undergo a five‐step maturation process, concurrent with significant morphological changes. At stage I and II, gametocytes are morphologically indistinguishable from the asexual stages, although *in Plasmodium falciparum* the IMC appears restricted to a single spine on one side of the parasite. From stage II to IV they obtain a characteristic crescent shape before rounding off in stage V (Sinden, 1982; Kono *et al*., 2012). This maturation is associated with the formation of a three membrane structure around the whole periphery of the cell (Sinden *et al*., 1978; Sinden, 1982). From the spine structure, the nascent IMC appears to extend initially along one side of the *P. falciparum* gametocyte, followed closely by microtubule deposition beneath the IMC and then recruitment of actin‐myosin motor components as confirmed using both established (PfGAP50, PfISP1) and novel (PF3D7_0525800) markers of the IMC (Dearnley *et al*., 2012; Kono *et al*., 2012; Poulin *et al*., 2013). Interestingly, the *Plasmodium*‐specific IMC‐resident protein MAL13P1.228 did not follow this localization, instead forming a lattice covering the stage III gametocyte which was maintained throughout maturation (Kono *et al*., 2012).

After being taken up by the mosquito, gametocytes mature into male or female gametes which fuse, forming a zygote which then differentiates into a motile ookinete, where the IMC again plays a key role in the development and maturation (Poulin *et al*., 2013). In *Plasmodium gallinaceum* and *P. berghi* ookinetes, as for merozoites, the IMC is derived from one flattened vesicle (Meszoely *et al*., 1982; Raibaud *et al*., 2001). A number of known IMC‐resident proteins including PfGAPM1 and the alveolin PF3D7_0525800 (Bullen *et al*., 2009; Kono *et al*., 2012) as well as components of the actin‐myosin motor complex have been localized to the ookinete IMC in *P. falciparum* (Dessens *et al*., 1999), confirming the similarity in make‐up to other life cycle stages. At this stage, the alveolins PbIMC1b and PbIMC1h are important in maintaining the ookinete morphology. Deletion of either protein resulted in a similar phenotype with abnormal ookinete morphology and motility, leading to a reduction in infectivity. Deletion of PbIMC1h also resulted in abnormal sporozoite morphology and a number of defects in *in vivo* infection. Interestingly, double knockout of PbIMC1b and PbIMC1h did not further alter ookinete shape, demonstrating that other proteins are also required in maintenance of ookinete shape and stability (Tremp and Dessens, 2011). The double mutant also suggested that the reduction in gliding motility was not due to the altered morphology of the ookinete, as the shape of the parasite remained similar while motility was further reduced (Tremp and Dessens, 2011). This suggests a functional link between IMC stability and the gliding machinery. Interestingly, PbIMC1h appears to be a close homologue of TgIMC3 (Tremp and Dessens, 2011) which is seen concentrated on daughter buds in *T. gondii*. However, the function of TgIMC3 in *T. gondii* remains unknown (Gubbels *et al*., 2004; Anderson‐White *et al*., 2011).

The structure of the IMC appears similar between ookinetes and other life stages; however, some differences have recently become apparent. PfISP1 is seen localized to the periphery in late gametocytes; however, in ookinetes it moves to the apical tip while PfISP3 maintains its peripheral localization (Poulin *et al*., 2013), demonstrating the existence of IMC subcompartments within the ookinete. Also restricted to ookinetes, the interaction of the IMC with subpellicular microtubules appears reliant on the activity of the phosphatase PbPPKL while expression of this protein is absent in most other life stages (Guttery *et al*., 2012; Philip *et al*., 2012). The metamorphosis from ookinete to oocyst recalls the structural transformation of sporozoites to trophozoite, the slender zoite first bulges then retracts the apical and basal ends, becoming spherical (Carter *et al*., 2007). The ookinete to oocyst transformation is also associated with loss of the IMC, but it is not known if this is via the same mechanism as described for sporozoite‐to‐trophozoite transformation (Jayabalasingham *et al*., 2010). Imaging using known markers of the IMC such as GAP50 could help dissect this process in more detail.

Interestingly, the shape changes during gametocyte maturation and ookinete transformation are not driven by the actin‐myosin motor but appear instead to be due to the formation of the IMC and subpellicular microtubules (Sinden, 1982; 1983; Kumar *et al*., 1985; Dearnley *et al*., 2012). Supporting this hypothesis, knockout of the key motor proteins MyoA and MTIP did not affect the formation or morphology of ookinetes, although were required for gliding motility (Sebastian *et al*., 2012), suggesting that formation and break‐down of the IMC is an important driver in the *Plasmodium* life cycle.

## Biogenesis of the inner membrane complex in *T**. gondii*

Due to the difficulty of obtaining the sexual stages of *T. gondii*, most work has been performed in asexual tachyzoites and comparatively little is known about other life cycle stages. However, cell division in the asexual form has been extensively studied, in part due to the ease of genetic manipulations in this parasite. In *T*. *gondii*, alveolins vary in localization and expression profiles through cellular division, leading to the definition of separate classes of IMC proteins, hinting at specific roles through cell division (Anderson‐White *et al*., 2011). The role and localization of these proteins during cell division has recently been extensively reviewed (Anderson‐White *et al*., 2013; Francia and Striepen, 2014) and so will not be described in detail here.

At the present time, no alveolin proteins have been disrupted in *T. gondii* and so any potential functional redundancy within this family is currently unknown. However, the IMC‐associated protein TgPhIL1 has been deleted, resulting in viable parasites which were shorter and wider than the wild type, although with no observable ultrastructural IMC defect (Barkhuff *et al*., 2011). These mutants replicated normally, but had subtly impaired motility and a fitness defect in mixed infection (Gilk *et al*., 2006; Barkhuff *et al*., 2011; Leung *et al*., 2014). This subtle phenotype recalls the deletion of *Plasmodium* alveolins which have relatively minor, stage‐specific effects. It will be interesting to see the results of deletion of the alveolin proteins in *T. gondii* and if any specific effects can be seen during cell division.

**Figure 1:**
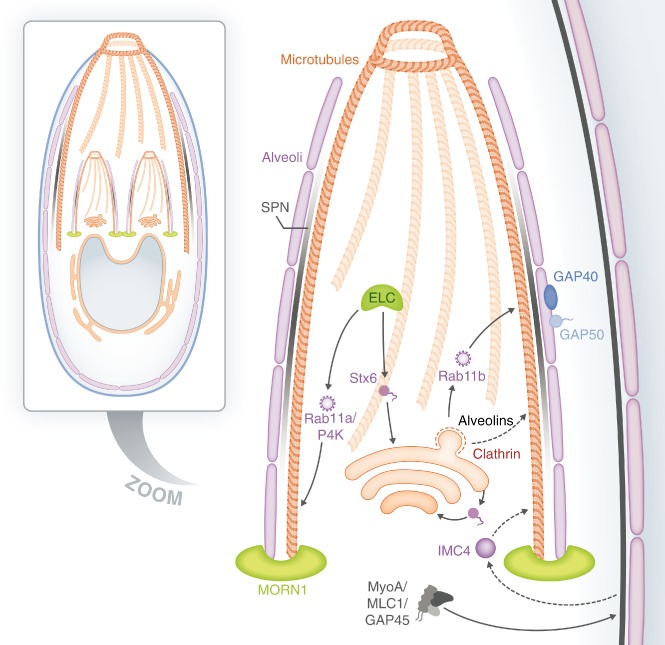
Overview of trafficking involved in IMC biogenesis in *T**. gondii*. See text for details, dashed arrows indicate unknown pathways. SPN, subpellicular network; MT, microtubules; P4K, phosphoinositide‐4‐OH‐kinase; Stx6, Syntaxin 6.

Vesicular trafficking in Apicomplexa is only just beginning to be understood (for a recent review see Tomavo *et al*., 2013); however recently several trafficking factors have been characterized which appear to have a role in IMC biogenesis (Fig. 1). The IMC of both *T*. *gondii* and *Plasmodium* is known to be constructed from clathrin‐coated vesicles derived from the ER‐Golgi secretory pathway (Bannister *et al*., 2000; Gordon *et al*., 2008; Yeoman *et al*., 2011; Pieperhoff *et al*., 2013). These ultrastructural observations were recently supported when overexpression of a dominant‐negative clathrin heavy chain construct in *Toxoplasma* (TgCHC1) was shown to lead to a number of defects, including a lack of microneme and rhoptry formation and a block in IMC biogenesis (Pieperhoff *et al*., 2013). Trafficking of IMC‐targeted vesicles is dependent on the highly conserved, apicomplexan‐specific, small GTPase TgRab11b (Agop‐Nersesian *et al*., 2010). In *T*. *gondii*, overexpression of a dominant‐negative TgRab11b construct resulted in disorganization of the daughter cell IMC (Agop‐Nersesian *et al*., 2010). This resulted in non‐viable parasites, demonstrating that recruitment of the alveoli and the SPN to the daughter scaffold is dependent on trafficking via Rab11b. A similar phenotype was observed for the actin‐like protein TgALP1, suggesting that this protein is also involved in IMC biogenesis (Gordon *et al*., 2008; 2010), although how this protein functions in reference to the IMC in currently unknown. Another trafficking factor, the recently characterized SNARE TgStx6, may also play a role in trafficking to the IMC (Jackson *et al*., 2013). TgStx6 is involved in retrograde transport between the endosomal like compartment (ELC) and Golgi and potentially has a role in maintaining Golgi organization (Jackson *et al*., 2013). However, due to the pleiotropic effects of TgStx6 overexpression, the mechanism is not yet clear.

Interestingly, while a proportion of TgIMC4 does appear to be recycled from the mother (Hu *et al*., 2006), TgIMC1 is not scavenged from the mother IMC, but rather is generated *de novo* (Hu *et al*., 2002; Mann *et al*., 2002). It is not currently known how this is trafficked or if any of the other alveolin proteins are recycled. At the end of budding, TgIMC1 is processed and the daughter IMC becomes a rigid, supporting structure (Mann *et al*., 2002) which is then enveloped by the mother cell's plasma membrane (Sheffield and Melton, 1968). It would be interesting to determine if this increased rigidity is due to the processing of TgIMC1 or the incorporation of new alveolins into the daughter SPN.

Later in budding, another apicomplexan‐specific, small GTPase named TgRab11a has been shown to be essential in IMC formation. Expression of a dominant‐negative TgRab11a construct resulted in a block in the later stages of cytokinesis in *T. gondii* while this gene was essential in *P. falciparum* (Agop‐Nersesian *et al*., 2009). Interestingly a phosphatidylinositol‐4‐OH kinase (P4K) also appears involved in Rab11a‐mediated vesicular trafficking. Blocking the activity of P4K in *Plasmodium* resulted in a late block in cytokinesis in a very similar manner to that observed in *T. gondii*, suggesting a functional link between these pathways in late stages of parasite budding (Agop‐Nersesian *et al*., 2009; McNamara *et al*., 2013).

The membrane‐localized protein MORN1 also appears to play an important role in IMC biogenesis in both *T*. *gondii* and *Plasmodium* (Gubbels *et al*., 2006; Ferguson *et al*., 2008). TgMORN1 is quickly recruited to the edge of the growing IMC, where it is thought to be important in the interaction between IMC and microtubules (Gubbels *et al*., 2006; Hu *et al*., 2006). The function (or functions) of MORN1 is still under debate; however, it is essential in cytokinesis as conditional deletion of TgMORN1 resulted in a defect in basal complex assembly, leading to the formation of crippled, multi‐headed parasites (Heaslip *et al*., 2010; Lorestani *et al*., 2010).

## The role of the IMC in parasite motility

One of the important roles of the IMC is to act as an anchor for the actin‐myosin motor complex which has an important role in parasite motility and invasion (Dobrowolski and Sibley, 1996; Opitz and Soldati, 2002; Andenmatten *et al*., 2013; Bargieri *et al*., 2013). This motor was first characterized in *T*. *gondii* and is well conserved across the Apicomplexa (Baum *et al*., 2006; Jones *et al*., 2006). Interestingly, although *Plasmodium* merozoites are immotile (Pinder *et al*., 2000), the motor complex remains important in erythrocyte invasion (reviewed in Farrow *et al*., 2011). The motor complex consists of the atypical myosin MyoA, its light chains MLC1 (named MTIP in *Plasmodium*) and ELC1 and the glideosome‐associated proteins GAP40, GAP45 and GAP50 (Herm‐Gotz *et al*., 2002; Bergman *et al*., 2003; Gaskins *et al*., 2004; Frenal *et al*., 2010; Nebl *et al*., 2011). This complex interacts with the glycolytic enzyme aldolase, actin, and transmembrane proteins of the TRAP family (Sultan *et al*., 1997; Jewett and Sibley, 2003; Huynh and Carruthers, 2006).

During cell division, MyoA, MLC1/MTIP and GAP45 are translated and form a complex in the cytoplasm (Gaskins *et al*., 2004; Rees‐Channer *et al*., 2006). In both *Toxoplasma* and *Plasmodium*, GAP45 is phosphorylated by calcium‐dependent kinases (Gilk *et al*., 2009; Nebl *et al*., 2011; Ridzuan *et al*., 2012; Thomas *et al*., 2012). However in *T. gondii*, but not *Plasmodium*, GAP45 must then be dephosphorylated before the assembly of the motor complex (Gaskins *et al*., 2004; Rees‐Channer *et al*., 2006; Gilk *et al*., 2009; Ridzuan *et al*., 2012; Thomas *et al*., 2012). The importance of GAP45 in maintaining the close association of the IMC to the plasma membrane is highlighted by recent studies demonstrating that deletion or ablation of this gene resulted in detachment of the IMC from the plasma membrane, in a similar manner to alpha toxin (Wichroski *et al*., 2002; Sebastian *et al*., 2012; Egarter *et al*., 2014). Once constructed in the cytoplasm, the complex is trafficked to the IMC, potentially via TgRab11a. However, the previous model whereby this complex binds directly to Rab11a via MyoA (Agop‐Nersesian *et al*., 2009) is now known to be incorrect, as deletion of TgMyoA or TgMLC1 does not affect IMC biogenesis or the localization of other components of the motor (Andenmatten *et al*., 2013), confirming results derived in *P. berghei* (Sebastian *et al*., 2012).

GAP50 is a transmembrane protein inserted directly into the alveolar membrane (Gaskins *et al*., 2004; Bosch *et al*., 2012). Supporting its function as the anchor of the motor complex, GAP50 was shown to be immobilized in detergent‐resistant regions of the IMC membrane, independent of direct interaction with proteins or microtubules, while GAP45 can freely diffuse (Johnson *et al*., 2007; Yeoman *et al*., 2011). In order to be targeted correctly, and to interact with the other motor complex proteins, GAP50 requires glycosylation at the amino‐terminus (Fauquenoy *et al*., 2011). Although the function of GAP50 has been initially characterized, the role of the 7‐transmembrane protein GAP40 remains unclear. GAP40 interacts with the components of the motor complex and is also present in early daughter cells at the same time as GAP50 (Frenal *et al*., 2010; Fauquenoy *et al*., 2011) and so may also have a role in anchoring the motor complex. Future studies are required to determine the function of GAP40 and the requirement for these two transmembrane proteins in motility and IMC formation.

Interestingly, disruption of IMC biogenesis can be demonstrated when the tail of the actin‐myosin motor protein TgMyoA is overexpressed in *T. gondii* (Agop‐Nersesian *et al*., 2009). It is known that deletion of MyoA or its light chain MLC1/MTIP does not affect IMC biogenesis in *T. gondii* or *Plasmodium* (Sebastian *et al*., 2012; Andenmatten *et al*., 2013) demonstrating that MyoA is not directly required for IMC biogenesis. This suggests that overexpression of the tail results in an indirect effect on IMC biogenesis, possibly through sequestering MyoA binding partners away from their site of action. The transmembrane proteins GAP40 and GAP50 would be interesting candidates in this hypothesis as these proteins are inserted into the IMC early in daughter cell development (Gaskins *et al*., 2004; Frenal *et al*., 2010). Interestingly, it has recently been shown that PfGAP45 is required for ookinete formation and shape change in *Plasmodium* (Sebastian *et al*., 2012), demonstrating that the motor complex has a role in both motility and IMC biogenesis.

In summary, the IMC is a fascinating, dynamic structure with known roles in parasite structure, division, morphogenesis and motility. By using the now well‐established genetic tools in *Plasmodium* and *T. gondii*, the functions of individual proteins are now adding to the early observational studies and allowing a much clearer understanding of the functions of this structure. Future studies will continue efforts to dissect the role of individual proteins in the construction and maintenance of the IMC.
